# Study of Flexural Response in Strain Hardening Cementitious Composites Based on Proposed Parametric Model

**DOI:** 10.3390/ma12010113

**Published:** 2018-12-31

**Authors:** Zhanfeng Qi, Zhiyi Huang, Hui Li, Wenhua Chen

**Affiliations:** College of Civil Engineering and Architecture, Zhejiang University, Hangzhou 310000, China; qizhanfeng@zju.edu.cn (Z.Q.); 21612089@zju.edu.cn (H.L.); 11812061@zju.edu.cn (W.C.)

**Keywords:** strain hardening cementitious composites, linear constitutive model, moment-curvature response, shear deflection

## Abstract

Strain hardening cementitious composites (SHCCs) are widely used in projects due to their excellent deformation resistance and large energy absorption capacity. However, determining tensile strain capacity is still a challenge for engineers. The current popular approach is to use inverse methods to predict the tensile behavior of SHCCs, such as the UM method (Qian and Li) and the JCI (Japan Concrete Institute) method. The key to these two approaches is to acquire the exact relationship between the bending and the uniaxial response. In this paper, a reasonable linear constitutive model of the SHCCs is modified. Initially, the moment-curvature diagrams are discussed by material parameters. The results reveal that the moment-curvature response is quite sensitive to the variations in the parameter of transition strain α, post-cracking tensile stiffness η, and strain softening stiffness μ, however, for the compressive parameters, the moment-curvature responses influence on flexural behavior is insignificant. Moreover, the load-deflection curve in the mid-span of SHCC, based on the consideration of shear effect, is simulated under a four-point bending test (FPBT). The results show a remarkable consistency with the experimental data when compared to the previous simulations. It is expected that this modified method can enhance an accurate program in order to obtain the tensile capacity.

## 1. Introduction

The weak-tension nature of traditional concrete and cement products is a primary attribute that has promoted the growth of modern design concepts regarding fiber-reinforcement cementitious composites. A class of strain hardening fiber reinforced cementitious composites featuring high ductility and moderate fiber content was designed by Li et al. in 1995 [1]. Over time, great strides have been made in developing the strain hardening cementitious composites [2,3,4]. Currently, the strain hardening behavior, regarding the ductile post-peak softening, has demonstrated that a non-catastrophic failure occurs as it is reinforced by short random fibers, which has provided a new possible solution for some infrastructures needing to resist external force and deformation. Of note, the emergence of ECC (engineered cementitious composites) materials with a moderate fiber content makes strain hardening cementitious composites (SHCCs) economical and feasible in engineering [5,6]. In the last decade, engineers have begun to use the ECC for the repair of aged infrastructure [7,8] such as bridge-deck links [9,10] and damping elements [11], while others have used the ECC to reinforce concrete beams for rehabilitation in projects [12,13]. In particular, the use of local cost-effective ingredients like polyvinyl alcohol fiber, fly ash, and local aggregate makes it more attractive to engineers [14,15,16,17]. Despite the success mentioned above, there are still some difficulties for engineers in determining the specific quantification of its tensile capacity with relatively small variation [18].

**Figure 1:**
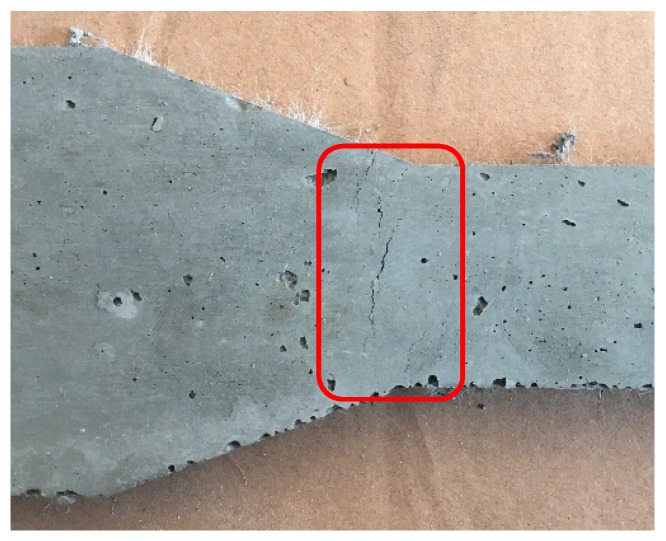
Initial cracks in a specimen caused by shrinkage stress.

Several approaches have been theoretically and experimentally implemented and can be classified into two patterns: (i) the direct assessments; and, (ii) the indirect assessments. The former is carried out by using uniaxial tensile tests (UTT) [19,20] but the testing process is sensitive to several factors such as sample imperfections, machine stiffness, slippage, stress concentration, and cracks, caused by shrinkage stress, as shown in Figure 1. Therefore, the indirect approach, which refers to the four-point bending test (FPBT), is more attractive to engineers. Such options include the JCI (Japan Concrete Institute) method proposed by the Japan Concrete Institute [21,22,23,24]; the DTU (Technical University of Denmark) method attempted by the Technical University of Denmark [25,26]; and the UM (Qian and Li ) method developed by the University of Michigan [27,28].

Admittedly, many scholars have done outstanding work on the three methods mentioned above. However, further simplification and accuracy are necessary to make the FPBT widely accepted for the quality control of SHCCs. For example, the JCI method requires several linear variable displacement transducers (LVDTs) to measure the beams curvature and it is not convenient to implement installation for the field conditions. On the other hand, the inverse process of the JCI method is equipped with complex calculations, such as solving the cubic equation. Regarding the DTU method, it has presented a consistency between the predicted load-deflection curves and the FEM analysis, however, no direction with the UTT results have been made. The UM method, by comparison, has been more appealing to engineers due to its simple test equipment and linear interpretation procedure [27,28].

**Figure 2:**
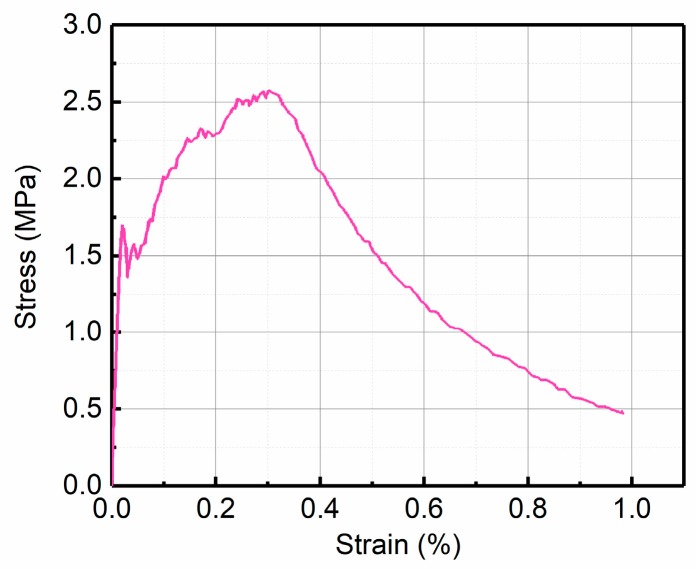
The stress–strain behavior of the SHCC in uniaxial tension.

Despite the UM method having been proven to improve the predicted accuracy of the system [27,28], there are still two crucial links to focus on. The first link is the uniaxial constitutive relation, such as the model proposed by Maalej and Li [29]. This model presented agreement between the prediction and the experiment stress-deflection for ECC but the tension model was incomplete for the SHCCs with some significant post-failure responses, as shown in Figure 2. 

The second link is the relationship between mid-span deflection and curvature, such as the load point deflection expression used by Qian and Li [27], as given by:

(1) $$u=\frac{15L^{2}}{162\rho }+\frac{L^{2}}{4\rho }{\left(\frac{h}{L}\right)}^{2}$$

where the second term of the above formula is caused by shear deflection. The size of specimens used by Qian and Li [27] was 51 mm in height and 305 mm in span length with (*h*/*L*)^2^ = (51/305)^2^ ≈ 0.028. This indicates that the second term is at least an order of magnitude smaller than the first term and, therefore, only the first term is considered. However, in engineering the sizes of 100 mm in height and 300 mm in span length [30]), and 101.6 mm in height and 304.8 mm in length span (ASTM standards [31]) are also commonly utilized with (*h*/*L*)^2^ = (100/300)^2^ = (101.6/304.8)^2^ ≈ 0.1. As the curvature of the material increases the second term in Equation (1) becomes more highly significant.

Keeping these considerations in mind, this paper will be the first to study the moment-curvature response of the SHCCs based on the modified uniaxial tension model. By considering the shear effect, a simulation is implemented to predict the four-point bending response. Simultaneously, the prediction results here will be compared with previous results obtained [27,29] which aims to develop a more reasonable and complete program for using the UM method.

## 2. Research Significance

This paper focuses on optimizing a method of analysis for deriving the moment-curvature response and the load-deflection relationship. Initially, the normalized parameters from the proposed model are used to define the position of the neutral axis, internal bending moment, and curvature in flexural beams so that the influence on the SHCC material behavior is independent from the specimen dimensions. The results can help engineers to understand the properties of the SHCCs and to assist in the design of targeted SHCC materials. The work of this article will make the UM method’s program more suitable and able to be applied universally.

## 3. Construction of Methods

### 3.1. Proposed Solutions for the Moment-Curvature Diagram of the SHCCs

**Figure 3:**
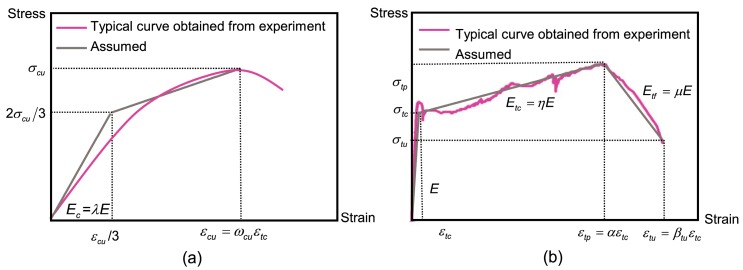
The proposed model of the SHCC in uniaxial tests, (**a**) stress-strain behavior of SHCC in uniaxial compression; (**b**) stress-strain behavior of SHCC in uniaxial compression.

Figure 3 presents the constitutive laws for the SHCC material used in this study. It can be observed from Figure 3a, that as the compressive curve linearly goes up to 2/3 of the ultimate stress σ_cu_, the strain reaches 1/3 of the maximum and, accordingly, the two calibration values in Figure 3a can be obtained on the basis of the equal energy principle [32]. Figure 3b demonstrates the proposed constitutive model with the descending branch of the SHCCs. The linear elastic range is defined by an initial tensile modulus E, the transition section is defined by a post-cracking modulus *E*_tc_, and the descending section is defined by a softening modulus *E*_tf_. In addition, there are three main parameters to be introduced: (i) the cracking tensile strain ε_tc_ which is an essential parameter for the SHCC material; (ii) the strain corresponding to peak stress ε_tp_; and, (iii) the termination strain ε_tu_. The parameters η and μ, as shown in Figure 3b, can be set as a positive or negative value to simulate the different strain tendencies. A negative value was set in this work due to the strain-hardening behavior. The correlation of stresses and strains for the compression and tension can be expressed as:

(2) $$\sigma_{c}\left(\varepsilon_{c}\right)=\left\{ \begin{array}{ll} E_{c}\varepsilon_{c} & 0\leq \varepsilon_{c}\leq \varepsilon_{\mathrm{cu}}/3\\ \frac{1}{3}E_{c}\varepsilon_{\mathrm{cu}}+\frac{\lambda E}{4}(\varepsilon_{c}-\frac{1}{3}\varepsilon_{\mathrm{cu}}) & \varepsilon_{\mathrm{cu}}/3<\varepsilon_{c}\leq \varepsilon_{\mathrm{cu}} \end{array}\right.$$

(3) $$\sigma_{t}\left(\varepsilon_{t}\right)=\left\{ \begin{array}{ll} E\varepsilon_{t} & 0\leq \varepsilon_{t}\leq \varepsilon_{\mathrm{tc}}\\ E\varepsilon_{\mathrm{tc}}+E_{\mathrm{tc}}{(\varepsilon }_{t}{-\varepsilon }_{\mathrm{tc}}) & \varepsilon_{\mathrm{tc}}<\varepsilon_{t}\leq \varepsilon_{\mathrm{tp}}\\ E\varepsilon_{\mathrm{tc}}+E_{\mathrm{tc}}(\varepsilon_{\mathrm{tp}}{-\varepsilon }_{\mathrm{tc}})+E_{\mathrm{tf}}(\varepsilon_{t}{-\varepsilon }_{\mathrm{tp}}) & \varepsilon_{\mathrm{tp}}<\varepsilon_{t}\leq \varepsilon_{\mathrm{tu}} \end{array}\right.$$

where the stresses and strains are denoted by σ_c_, σ_t_, ε_c_ and ε_t_, respectively. Several control parameters, as shown in Figure 3, are normalized by ε_tc_ and *E*, as expressed by:

(4) $$\alpha =\frac{\varepsilon_{\mathrm{tp}}}{\varepsilon_{\mathrm{tc}}}{;\;\beta }_{\mathrm{tu}}=\frac{\varepsilon_{\mathrm{tu}}}{\varepsilon_{\mathrm{tc}}}{;\;\omega }_{\mathrm{cu}}=\frac{\varepsilon_{\mathrm{cu}}}{\varepsilon_{\mathrm{tc}}}$$

(5) $$\lambda =\frac{E_{c}}{E};\;\eta =\frac{E_{\mathrm{tc}}}{E};\;\mu =\frac{E_{\mathrm{tf}}}{E}$$

By adopting the Bernoulli’s theory of plane section, the remaining plane and the assumption of a linear distribution of strain across the depth meant that the strain at the top fiber ε_ctop_ was deduced by the single independent variable ε_tbot_, as expressed by:

(6) $$\beta =\frac{\varepsilon_{\mathrm{tbot}}}{\varepsilon_{\mathrm{tc}}};\;\omega =\frac{\varepsilon_{\mathrm{ctop}}}{\varepsilon_{\mathrm{tc}}}$$

The position of the neutral axis was derived by the parameter *k*:

(7) $$\frac{\varepsilon_{\mathrm{ctop}}}{\varepsilon_{\mathrm{tbot}}}=\frac{kd}{d-kd}\;\mathrm{or}\;\omega =\frac{k}{1-k}\beta$$

**Figure 4:**
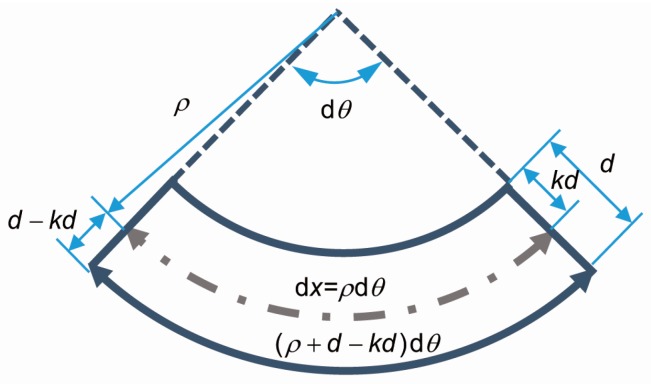
The curvature of a differential beam element.

The beam curvature can be derived from Figure 4:

(8) $$\varepsilon_{\mathrm{tbot}}=\frac{(\rho +d-kd)d\theta -\rho d\theta }{\rho d\theta }=\frac{d-kd}{\rho }$$

(9) $$\phi =\frac{1}{\rho }=\frac{\varepsilon_{\mathrm{tbot}}}{d-kd}$$

By substituting Equations (4)–(7) into Equations (2) and (3), the normalized stress–strain relationship can be expressed as:

(10) $$\frac{\sigma_{c}\left(\omega \right)}{E\varepsilon_{\mathrm{tc}}}=\left\{ \begin{array}{ll} \lambda \omega & 0\leq \omega \leq \omega_{\mathrm{cu}}/3\\ \frac{1}{3}{\lambda \omega }_{\mathrm{cu}}+\frac{\lambda }{4}(\omega -\frac{1}{3}\omega_{\mathrm{cu}}) & \omega_{\mathrm{cu}}/3<\omega \leq \omega_{\mathrm{cu}} \end{array}\right.$$

(11) $$\frac{\sigma_{t}\left(\beta \right)}{E\varepsilon_{\mathrm{tc}}}=\left\{ \begin{array}{ll} \beta & 0\leq \beta \leq 1\\ 1+\eta (\beta -1) & 1<\beta \leq \alpha \\ 1+\eta (\alpha -1)+\mu (\beta -\alpha ) & \alpha <\beta \leq \beta_{\mathrm{tu}} \end{array}\right.$$

**Figure 5:**
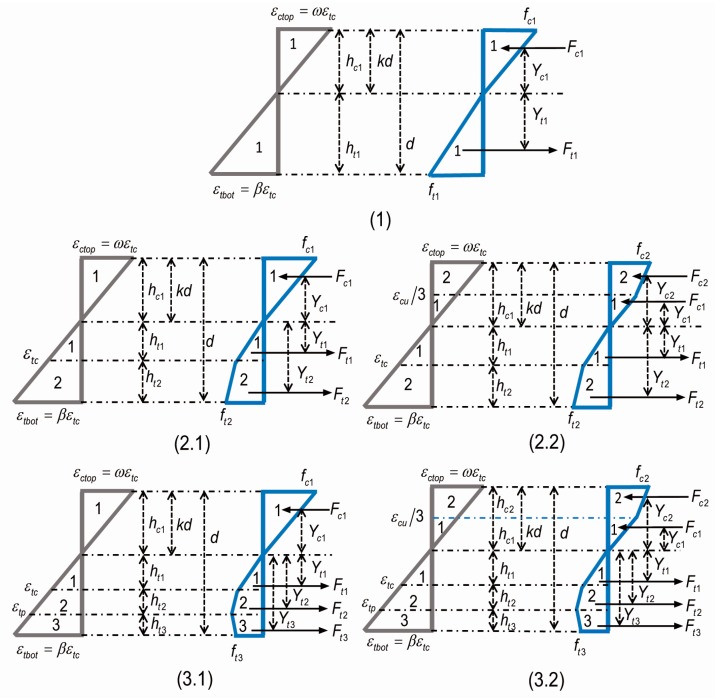
The strain and stress distributions for the different stages with applied normalized tensile strain at the bottom fiber (β).

Figure 5 presents the distribution of stress and strain based on proposed constitutive models. Clearly, three different stages could be divided according to the parameter β: (i) stage 1 represents the elastic phase with 0 < β ≤ 1; (ii) stage 2 represents the hardening phase with 1 < β ≤ α; and, (iii) stage 3 represents the softening phase with α < β < β_tu_. These stages are shown in Table 1. However, it should be noted that there were two possible situations for stages 2 and 3 which depended upon the condition of the highest-compression fiber: (i) the elastic (0 ≤ ω ≤ ω_cu_/3); or, (ii) the plastic (ω_cu_/3 ≤ ω ≤ ω_cu_) state.

The values of *h_i_* and *Y_i_* in Figure 5 can be obtained by explicit integration across the thickness of the compression zone. Taking the example of stage 1 (the force component *F*_c1_ and its lever arm γ_c1_) the integration is expressed as:

(12) $$F_{c1}=b\int_{0}^{kd}\sigma_{c1}(z)dz;\;Y_{c1}=\frac{b}{F_{c1}}\int_{0}^{kd}\sigma_{c1}(z)zdz$$

(13) $$\frac{F_{c1}}{bdE\varepsilon_{\mathrm{tc}}}=\frac{\lambda \beta k^{2}}{2(1-k)};\;\frac{Y_{c1}}{d}=\frac{2k}{3}$$

where the parameter *z* is in the position of ε_c*i*_, calculated from the neutral axis. All heights and lever arms, as shown in Figure 5, were normalized with respect to beam depth *d*. By solving Equation (14) for static equilibrium in the horizontal direction of the cross section, the axis depth ratio *k* was obtained:

(14) $$\sum F_{i}=0\;(i\;\mathrm{represents}\;\mathrm{any}\;\mathrm{stage})$$

In general, the position of the neutral axis is at the centroid of the rectangular section for an isotropic material and thus, the initial value of *k* was assumed to be 0.5. The alternate value of *k* should lie within the range of 0 < *k* < 1. The moment *M_i_*, as shown in Table 2, at each stage is computed by using Equation (15):

(15) $$M_{i}=\sum F_{i}Y_{i}$$

Similarly, using Equations (16) and (17), the normalized process was implemented and the expressions for the moment combined with *k* are presented in Table 3.

(16) $$M_{i}=M_{i}^{\prime }M_{\mathrm{tc}};\;M_{\mathrm{tc}}=\frac{1}{6}bd^{2}E\varepsilon_{\mathrm{tc}}$$

(17) $$\phi_{i}=\phi_{i}^{\prime }\phi_{\mathrm{tc}};\;\phi_{\mathrm{tc}}=\frac{2\varepsilon_{\mathrm{tc}}}{d}$$

### 3.2. Crack-Localization Rules

**Figure 6:**
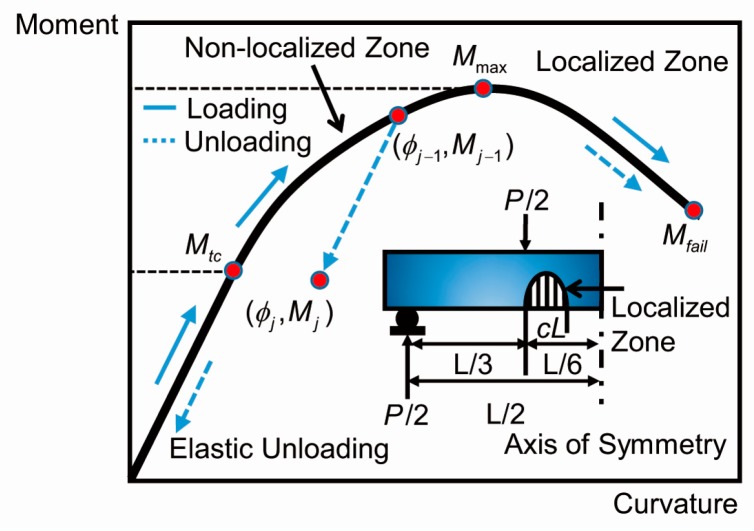
The moment-curvature response and the crack localization rules. Inset: schematic of a four-point bending test (only left half of the model is shown).

Figure 6 presents the schematics of the moment-curvature response with respect to the crack-localization rules for the loaded and unloaded steps. Once the loads on a sample exceed the flexural strength the load begins to fall. Meanwhile, simultaneously, there are two distinct regions evolving on the beam: (i) a localized zone; and, (ii) a non-localized zone. In general, the localized region, owing to the damage of cracking, has no capability to bear the load sequentially. Conversely, the non-localized region can bear the unloading as the deflection continues to increase [33] which causes a continuous increase in the deflection of the mid-span. 

The inset of Figure 6 shows the schematic diagram of a four-point bending test. It is observed that a localized zone occurs in the mid-span while the location of the crack is random. In order to describe the localized zone behavior, in terms of the stress-strain relationship instead of a stress-cracking response, it is assumed that the main cracks are smeared uniformly over the mid-section zone. The length of the smeared-crack zone is defined as *cL* and the range of *c* is 0 ≤ *c* ≤ 1/6, where *L* is the clear span. Assuming a default value of *c* = 1/6, this signifies that the cracks are smeared over the entire mid-span.

Dividing the moment-curvature response diagram, as shown in Figure 6, into three portions: (i) an ascending portion from 0 to *M*_tc_ which corresponds to the elastic response with a unique path of loading and unloading; (ii) a non-elastic ascending portion from *M*_tc_ to *M*_max_. Any point included in this portion of the curve indicates the beginning of unloading and the unloading stiffness is consistent with the elastic stiffness; and, (iii) the descending portion from *M*_max_ to *M*_fail_.

For each loading step the moment and the subsequent curvature along the entire length of the beam are calculated by solving the static balanced equation. Once the loading exceeds the maximum value, the curvature in the cracked region is based upon the descending portion from $M_{max}\;\mathrm{to}\;M_{fail}$. In the uncracked region, the curvature decreases linearly and elastically during unloading where $M<M_{tc}$. If the section load exceeds $M_{tc}$, the unloading curvature of the cracked area will be accompanied by a quasi-linear recovery path expressed as:

(18) $$\phi_{i}=\phi_{j-1}-\xi \frac{\left(M_{j-1}-M_{j}\right)}{EI}$$

where ϕ*_j_*_−1_ and *M_j_*_−1_ represent the previous state of the curvature and moment, respectively. Both ϕ*_i_* and *M_j_* are the current states. The unloading factor is 0 < ξ < 1, ξ = 1 and signifies unloading with initial elastic stiffness. When ξ = 0, this indicates no curvature recovery during unloading, which is the situation assumed in the present work.

### 3.3. Procedures to Obtain the Load-Deflection Behavior from a Four-Point Bending Test

The expression of the load point deflection for a beam with span length L is given by Equation (1) but the mid-span deflection is much easier to acquire through a material testing system (MTS). Therefore, it is necessary to obtain the expression at mid-span deflection. The procedure for the expression is as follows: (i) computing the moment and curvature of the given section and the material parameters by using Equations (16) and (17) as the parameter β increases; (ii) considering the model shown in Figure 6, the load capacity carried by the beam is *P* = 6*M*/*L*; (iii) for each loading step, the distribution of the moment and curvature were obtained through static equilibrium and crack-localization rules; and (iv) the deflection at mid span was determined by the classical beam theory of differential equations. When the bending stiffness is at a constant, the equation of deflection curve can be given by:

(19) $$M(x)=-EI\frac{d^{2}w_{f}}{dx^{2}}$$

The expression for the bending moment for each section is shown in Equation (20):

(20) $$M(x)=\left\{ \begin{array}{ll} \frac{Px}{2} & 0\leq x\leq L/3\\ \frac{PL}{6} & L/3\leq x\leq 2L/3\\ \frac{P}{2}(L-x) & \;2L/3\leq x\leq L \end{array}\right.$$

It is necessary to use the constraint condition for the support and continuous conditions for the displacement of the two adjacent sections of the beam to determine the integral constants. The result is expressed as:

(21) $$w_{f}(x)=\frac{1}{EI}(-\frac{PL}{12}x^{2}+\frac{PL^{2}}{12}x-\frac{PL^{3}}{324})\;(L/3\leq x\leq 2L/3)$$

Utilizing the physical relationship between the curvature and the bending moment is given by:

(22) $$\frac{1}{\rho (x)}=\frac{M(x)}{EI}$$

If the curvature is a constant along the beam, the flexural deflection at mid span for a beam, given a clean span *l*, is expressed as:

(23) $$w_{f}(\frac{l}{2})=\frac{23L^{2}}{216\rho }$$

In this study, a Poisson’s ratio of 0.2 [34] for the ECC material and a shear area of 5*bd*/6 are assumed in conformity with the Timoshenko theory. The expression for shear deflection is:

(24) $$w_{s}(\frac{l}{2})=\frac{1}{GA}\int Q(x)dx=\frac{12PL}{25Ebd}$$

**Figure 7:**
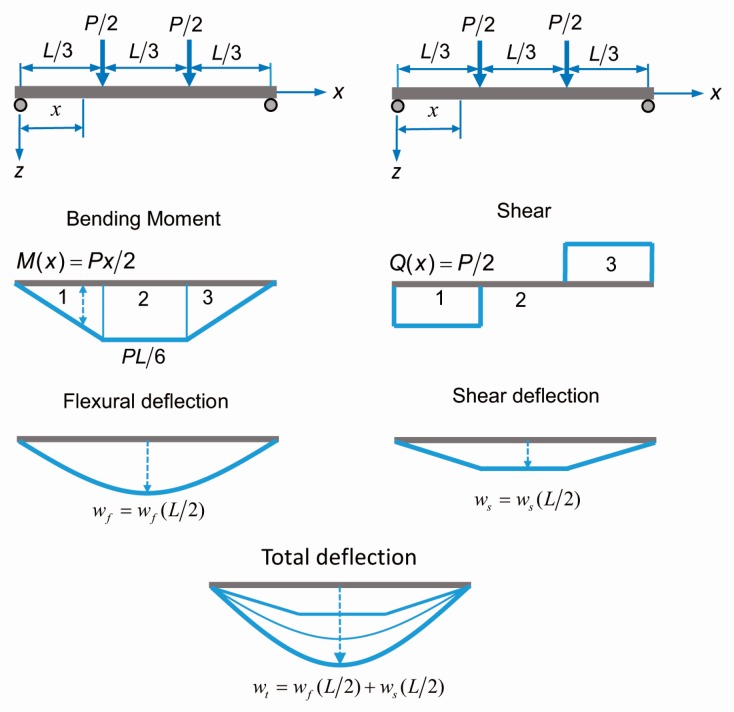
The procedure to obtain the load-total deflection in a four-point beading test.

By substituting p=6M/L and G=E/2(1+υ) into Equation (24) the total deflection shown in Figure 7 at the mid-span can then be expressed as:

(25) $$w_{t}=w_{f}+w_{s}=\frac{1}{\rho }L^{2}\left[\frac{23}{216}+\frac{6}{25}{\left(\frac{d}{L}\right)}^{2}\right]$$

**Figure 8:**
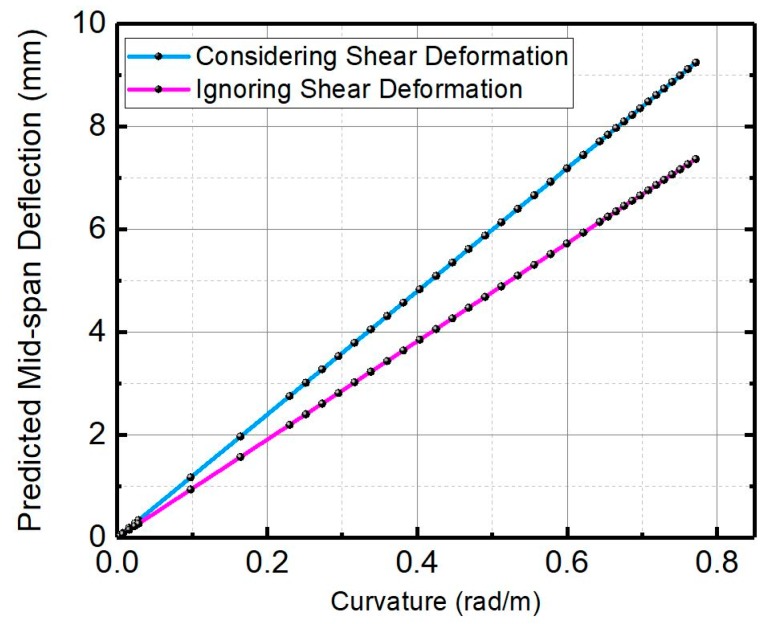
The relationship between the mid-span total deflection and the curvature.

In order to confirm the shear effect Equation (25) is plotted in Figure 8 with a size of 100 × 100 × 400 mm^3^. It is clear that the shear deflection has a significant influence on the total deflection, especially with the curvature increasing. Therefore, it is reasonable to consider the shear deformation for the SHCCs as the deflection-curvature is adopted for the UM method.

## 4. Parametric Study of Material Parameters

**Figure 9:**
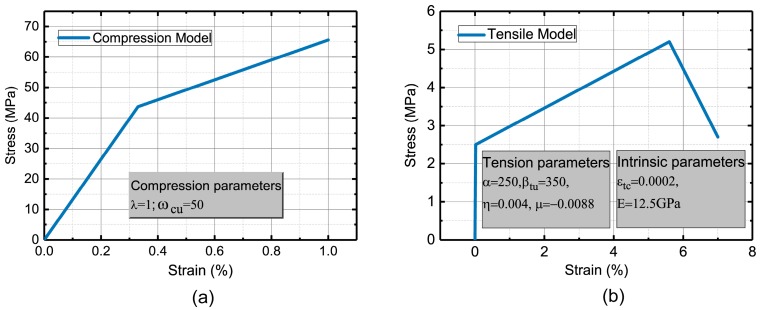
The typical curve obtained from the uniaxial experiment, (**a**) compression model; (**b**) tension model.

A series of basic experimental results from Reference [1] is to implement the study, however, the compressive and tensile parameters are presented in Figure 9a,b, respectively.

**Figure 10:**
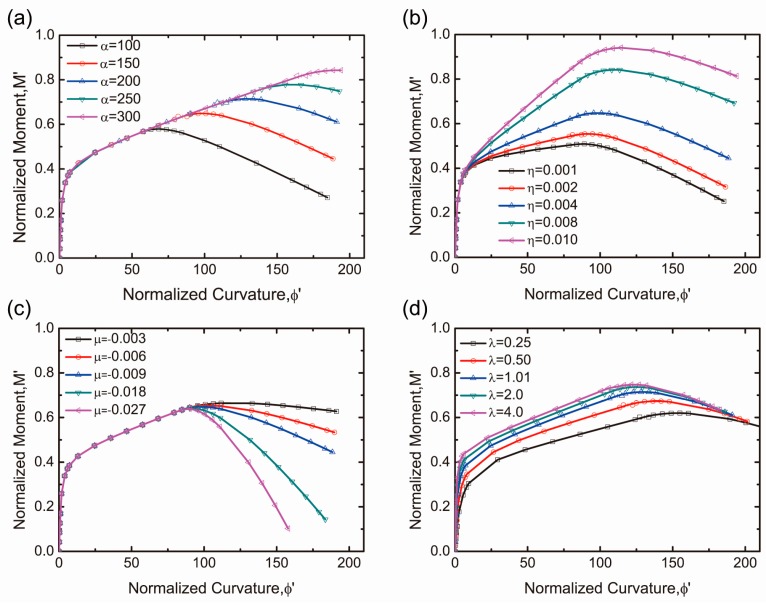

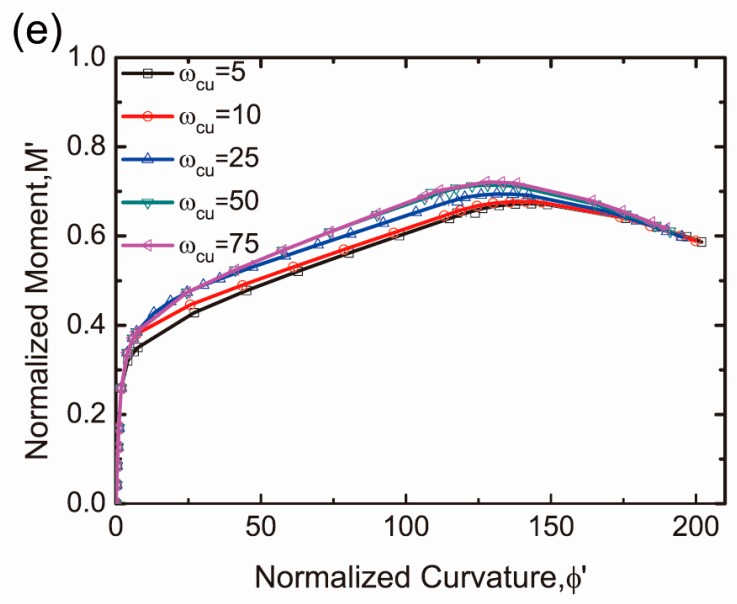
Parametric studies of the five material parameters on the normalized moment curvature response: (**a**) vary α; (**b**) vary η; (**c**) vary μ; (**d**) vary λ and (**e**) vary ω_cu_

Figure 10a–e shows the normalized moment-curvature responses with the parameters of α, η, μ, and λ with the influence of the tensile parameters discussed first. Figure 10a demonstrates that the parameter α has a significant effect on the peak strength and ductility. As the transition strain α increases, both flexural strength and ductility raise linearly. The impact on the parameter η is shown in Figure 10b. It is observed that the ultimate strength is quite sensitive to the variations in η but it differs from the influence of parameter α. When the value of η increases, its peak strength increases with an enhancing stiffness. Figure 10c demonstrates that the change in the μ value has little effect on the peak strength but has a significant effect on the post-peak response. Like the tensile parameters, the compressive materials λ and ω_cu_ are also studied in Figure 10d,e.

The parameter λ represents the ratio between the compression elastic modulus and the tension elastic modulus. As the ratio increases, the peak value increases while the ductility decreases slightly, as shown in Figure 10d. The effect of the ω_cu_ variation on the peak strength and ductility is demonstrated in Figure 10e. It is clear that the effect of ω_cu_ on the bending response is extremely small. Comparing this with the effect of tensile parameters, the influence caused by the change of compression parameters is relatively insignificant.

## 5. Simulation and Discussion

**Figure 11:**
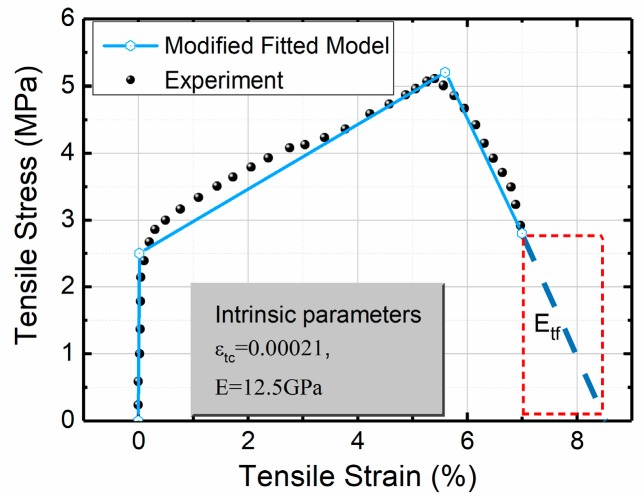
The fitted tension model of the SHCC used in this simulation.

The SHCCs employed in this study were polyethylene fibers with 2% volume fraction. The mix ratio was 1:0.75:0.1:0.02 by weight (cement: sand: silica fume: super plasticizer). The water to cement ratio was 0.3. All the parameters were determined by fitting the model from the uniaxial test result [29]. The average material properties were compressive strength *f*_c_ = 60 MPa; ω_cu_ = 55, α = 267, the post-crack stiffness η = 0.0041, the failure stiffness μ = −0.0144, and the ultimate tensile strain level β_tu_ = 333. The intrinsic parameters are illustrated in a solid box in Figure 11.

**Figure 12:**
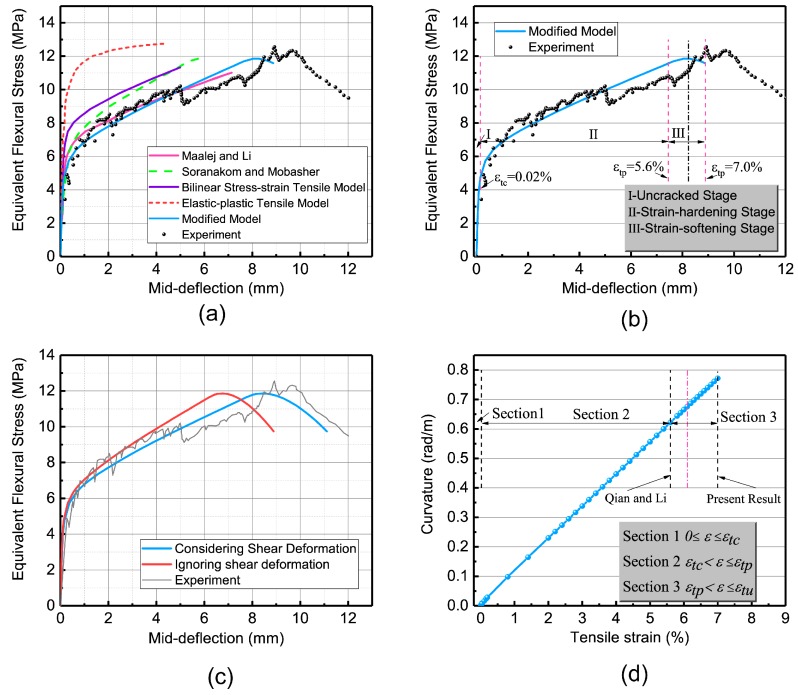
The simulation of a SHCC: (**a**) simulated and observed equivalent flexural stress vs. deflection responses; (**b**) predicted curve between moment and tensile strain; (**c**) simulated and observed equivalent flexural stress vs. deflection responses based on the shear effect; and (**d**) computed curvature vs. tensile strain.

The simulated result and the experimental data are presented in Figure 12a. Simultaneously, the previous results obtained by the JCI method [22,35] and the UM method [29] are compared. It is clear from Figure 12a that the predicted results through the JCI method used by Soranakom [22] and Suryanto [35] are exaggerated when compared with the experimental data, which is caused by the use of the ideal elastic-plastic model featuring the compressive and tensile behavior. The simulation through the UM method used by Qian and Li [27] cannot achieve the peak value as sufficiently when compared to the JCI method [35]. This is due to the fact that the post-peak response of the tensile model is not involved. It is clearly illustrated in Figure 12b that the flexural response is hardening while the uniaxial tension response is softening. Therefore, when the observed mid-span deflection is utilized to determine the uniaxial tensile strain the predicted results are amplified.

In addition, by assuming a complete tensile model, as shown by the dashed rectangle in Figure 11, the softening section of the bending response is also predicted in Figure 12c. The predicted curve displays agreement in all phases of the flexural behavior. Figure 12c depicts another meaningful simulation result based on the shear influence. By considering the shear deflection, this produces a greater consistency with the experimental data. The computed curvature is presented in Figure 12d. It is clear that as the tensile strain enters the softening stage (stage 3) the curvature of the beam is still increasing which also confirms that the post-peak response of tension has a significant impact on the flexural behavior of the SHCCs.

## 6. Summary and Future Work

This paper presents the solutions for generating load-deflection diagrams of SHCCs. The specific conclusions are drawn as follows:

- The parametric study reveals that the tensile parameters mainly dominate the flexural performance in the moment-curvature diagram. Specifically, the transition strain α and post-cracking tensile stiffness η have a direct influence on the peak flexural strength, and the failure stiffness μ has a vital impact on post-peak response. Compared to the tension parameters, the influence of compressive parameters on bending behavior is insignificant.
- The calculation of mid-deflection is affected by the shear effect which depends upon the ratio of specimen’s height to span length. Generally, when the specimen size is 100 × 100 × 400 mm^3^ or 76.2 × 101.6 × 355.6 mm^3^ the expression of deflection requires it to take the term into account caused by the shear deflection.
- The predicted load-deflection curve, based upon considering shear effect and post-peak response, presents reasonable consistency when compared with the experiment results under a four point-bending test, especially in the peak and softening portions. The results help to form a reasonable procedure for using the UM method which is utilized to determine the tensile behavior of the SHCCs accurately.

Although the present simulations show a good consistency with the experimental results, it is still not enough to put the UM method into application. This indicates that the deflection-tensile strain curve needs to be established with a large number of test data and this process is intended to be implemented in future work by the author.

## Figures and Tables

**Table 1:** The range of compression and tension for each stage of applied strain at the most tensile fiber (β).

Stage	Tension	Compression
1	1 < β ≤ 1	0 ≤ ω ≤ ω_cu_/3
2.1	1 < β ≤ α	0 ≤ ω ≤ ω_cu_/3
2.2	1 < β ≤ α	ω_cu_/3 ≤ ω ≤ ω_cu_
3.1	α < β ≤ β_tu_	0 ≤ ω ≤ ω_cu_/3
3.2	α < β ≤ β_tu_	ω_cu_/3 ≤ ω ≤ ω_cu_

**Table 2:** Equilibrium of the force and the internal moment for each stage of applied strain at the most tensile bottom fiber (β).

Stage	Force Equilibrium	Internal Moment
1	∑*F*_1_ = −*F*_c1_ + *F*_t1_	*M*_1_ = *F*_c1_*Y*_c1_ + *F*_t1_*Y*_t1_
2.1	∑*F*_2.1_ = −*F*_c1_ + *F*_t1_ + *F*_t2_	*M*_2.1_ = *F*_c1_*Y*_c1_ + *F*_t1_*Y*_t1_ + *F*_t2_*Y*_t2_
2.2	∑*F*_2.2_ = −*F*_c1_ − *F*_c2_ + *F*_t1_ + *F*_t2_	*M*_2.2_ = *F*_c1_*Y*_c1_ + *F*_c2_*Y*_c2_ + *F*_t1_*Y*_t1_ + *F*_t2_*Y*_t2_
3.1	∑*F*_3.1_ = −*F*_c1_ + *F*_t1_ + *F*_t2_ + *F*_t3_	*M*_3.1_ = *F*_c1_*Y*_c1_ + *F*_t1_*Y*_t1_ + *F*_t2_*Y*_t2_ + *F*_t3_*Y*_t3_
3.2	∑*F*_3.2_ = −*F*_c1_ − *F*_c2_ + *F*_t1_ + *F*_t2_ + *F*_t3_	*M*_3.1_ = *F*_c1_*Y*_c1_ + *F*_c2_*Y*_c2_ + *F*_t1_*Y*_t1_ + *F*_t2_*Y*_t2_ + *F*_t3_*Y*_t3_

**Table 3:** Solutions of the neutral-axis depth ratio and normalized moment, and the curvature for each stage of applied strain at the most tensile fiber (β).

Stage	*k*	*M′*
1	$k_{1}=\left\{ \begin{array}{ll} \frac{1}{2} & \mathrm{for}\;\lambda =1\\ \frac{-1+\sqrt{\lambda }}{-1+\lambda } & \mathrm{for}\;\lambda <1\;\mathrm{or}\;\lambda >1 \end{array}\right.$	$M_{1}^{\prime }=\frac{\beta \left[{\left(1-k_{1}\right)}^{3}+\lambda k_{1}{}^{3}\right]}{3(1-k_{1})}$
2.1	$\begin{array}{l} k_{21}=\frac{D_{21}-\sqrt{{\lambda \beta }^{2}D_{21}}}{D_{21}-{\lambda \beta }^{2}}\\ D_{21}=\eta \beta^{2}-2\eta \beta +\eta +2\beta -1 \end{array}$	$\begin{array}{c} M_{21}^{\prime }=\frac{C_{21}-3C_{21}k_{21}+3C_{21}k_{21}{}^{2}-(C_{21}+2\beta^{3}\lambda )}{6(k_{21}-1)\beta^{2}}\\ C_{21}=-2+(1-\beta )[3-\beta (-3+\eta )-{\eta +2\beta }^{2}\eta ] \end{array}$
2.2	$\begin{array}{l} k_{22}=\frac{4D_{22}+3({\beta \lambda \omega }_{\mathrm{cu}}-{\lambda \omega }_{\mathrm{cu}}{}^{2})-2\sqrt{{3\beta }^{2}\lambda D_{22}}}{4D_{22}-3(\beta^{2}{\lambda +2\beta \lambda \omega }_{\mathrm{cu}}{+\lambda \omega }_{\mathrm{cu}}{}^{2})}\\ D_{22}=-3+6\beta +3\eta -6\beta \eta +3\beta^{2}{\eta +\lambda \omega }_{\mathrm{cu}}{}^{2} \end{array}$	$\begin{array}{c} M_{22}^{\prime }=\frac{C_{22}-3C_{22}k_{22}+3(C_{22}+81\beta^{2}{\lambda \omega }_{\mathrm{cu}})k_{22}^{2}-(C_{22}-54\beta^{3}{\lambda +81\beta }^{2}{\lambda \omega }_{\mathrm{cu}})k_{22}^{3}}{648(k_{21}-1)\beta^{2}}\\ C_{22}=108-324\beta^{2}-108{\eta +324\beta }^{2}\eta -{216\beta }^{3}\eta -{19\lambda \omega }_{\mathrm{cu}}^{3} \end{array}$
3.1	$\begin{array}{l} k_{31}=\frac{-D_{31}-\sqrt{\beta^{2}\lambda D_{31}}}{-D_{31}+\beta^{2}\lambda }\\ D_{31}=-1+\eta -\alpha^{2}{\eta +\alpha }^{2}{\mu +\beta }^{2}\mu +\\ 2\beta [1+(\alpha -1)\eta -\alpha \mu ] \end{array}$	$\begin{array}{c} M_{31}^{\prime }=\frac{C_{31}+D_{31}-(3C_{31}+2E_{31})k_{31}+(3C_{31}+2E_{31})k_{31}^{2}-(C_{31}-{2\beta }^{3}\lambda )k_{31}^{3}}{6(1-k_{31})\beta^{2}}\\ C_{31}=2-3\alpha^{2}{+3\beta }^{2}{+3\alpha }^{2}\eta -{3\alpha }^{3}\eta -{3\beta }^{2}{\eta +3\alpha \beta }^{2}{\eta +2\alpha }^{3}\mu \\ -{3\alpha }^{2}{\beta \mu +\beta }^{3}\mu \\ E_{31}=-6\beta +6\alpha \beta +3\beta \eta -6{\alpha \beta \eta +3\alpha }^{2}\beta \eta \end{array}$
3.2	$\begin{array}{l} k_{32}=\frac{-4D_{32}-3({\beta \lambda \omega }_{\mathrm{cu}}-{\lambda \omega }_{\mathrm{cu}}^{2})-2\sqrt{3\beta^{2}\lambda D_{32}}}{-4D_{32}+3(\beta^{2}\lambda -{2\beta \lambda \omega }_{\mathrm{cu}}{+\lambda \omega }_{\mathrm{cu}}^{2})}\\ D_{32}=-3-3(\alpha^{2}-1)\eta +6\beta [1+(\alpha -1)\eta ]+\\ 3(\alpha -{\beta )}^{2}{\mu +\lambda \omega }_{\mathrm{cu}}^{2} \end{array}$	$\begin{array}{c} M_{32}^{\prime }=\frac{C_{32}-3C_{32}k_{32}-(C_{32}+18\beta^{3}\lambda -27\beta^{2}{\lambda \omega }_{\mathrm{cu}})k_{32}^{2}+3(C_{32}-9\beta^{2}{\lambda \omega }_{\mathrm{cu}})k_{32}^{3}}{216(k_{32}-1)\beta^{2}}\\ C_{32}=36+36(\alpha^{3}-1)\eta -108\beta^{2}[1+(\alpha -1)\eta ]-36{\left(\alpha -\beta \right)}^{2}(2{\alpha +\beta )\mu +\lambda \omega }_{\mathrm{cu}}^{3} \end{array}$
